# Filling the Gaps in the Pharmacy Workforce in Post-Conflict Areas: Experience from Four Countries in Sub-Saharan Africa

**DOI:** 10.3390/ijerph18158132

**Published:** 2021-07-31

**Authors:** Anabelle Wong, Kevin K. C. Hung, Mzwandile Mabhala, Justin W. Tenney, Colin A. Graham

**Affiliations:** 1 The Institute of Public Health, Charité—Universitätsmedizin Berlin, 10117 Berlin, Germany; anabellewong.hk@gmail.com; 2 Max Planck Institute for Infection Biology, 10117 Berlin, Germany; 3 Collaborating Centre for Oxford University and CUHK for Disaster and Medical Humanitarian Response (CCOUC), The Chinese University of Hong Kong, Hong Kong, China; cagraham@cuhk.edu.hk; 4 Accident and Emergency Medicine Academic Unit, The Chinese University of Hong Kong, Hong Kong, China; 5 Department of Public Health and Wellbeing, University of Chester, Chester CH1 4BJ, UK; a.mabhala@chester.ac.uk; 6 School of Pharmacy, West Coast University, Los Angeles, CA 92617-3040, USA; jtenney@westcoastuniversity.edu

**Keywords:** health workforce, pharmacy, post-conflict, health emergency and disaster risk management, universal health coverage

## Abstract

Background: While the pharmacy workforce is the third largest professional healthcare group worldwide, the pharmacy workforce landscape remains unclear in post-conflict areas in sub-Saharan Africa. Method: Key informants were selected for semi-structured interviews due to their role in providing pharmacy services in the selected country: the Central African Republic (CAR), the Democratic Republic of Congo (DRC), Ethiopia, and South Sudan. Transcripts from the interviews were anonymized, coded, and analyzed. Results: Nine participants were recruited (CAR: 2; DRC: 2; Ethiopia: 2; South Sudan: 3), and all except two were pharmacists. Conflict-specific challenges in pharmacy service delivery were identified as the following: unpredictable health needs and/or mismatched pharmaceutical supply, transport difficulties due to insecure roads, and shortage of pharmacy workforce due to brain drain or interrupted schooling. Barriers to health workforce retention and growth were identified to be brain drain as a result of suboptimal living and working conditions or remuneration, the perception of an unsafe work environment, and a career pathway or commitment duration that does not fit the diaspora or expatriate staff. Conclusion: To tackle the barriers of pharmacy health workforce retention and growth, policy solutions will be required and efforts that can bring about long-term improvement should be prioritized. This is essential to achieve universal health coverage and the targets of the sustainable development goals for conflict affected areas, as well as to “leave no one behind”.

## 1. Introduction

The goal of humanitarian response is to meet basic needs and to reduce future vulnerabilities to disasters [1]. Healthcare is a critical aspect in all stages of disasters and complex emergencies. Conflict areas tend to suffer from extensive violence and loss of life, massive displacements of people, widespread damage to societies and economies, and significant security risks for humanitarian relief workers [2].

Conflicts have caused massive displacements and widespread hunger around the world. In 2018, there were 52 conflicts in 36 different countries, causing 53,000 deaths [3]. The United Nations High Commissioner for Refugees (UNHCR) estimated that 70.8 million people were forcibly displaced due to conflicts, violence, persecution, or human rights violations [4]. The United Nations’ Food and Agriculture Organization (FAO) and World Food Program (WFP) in 2019 reported around 56 million people urgently need food and livelihood assistance across eight conflict zones around the world [5]. Worse still, long running crises can sever the health system of a country or region, leaving the affected areas with little to no capacity to cope with the surge in healthcare needs in emergencies such as outbreaks of infectious diseases and natural disasters.

Rebuilding of health services in these areas takes place with a backdrop of insecurity, as smaller scale conflicts may continue in the post-conflict stage. Weak management systems, damaged infrastructure, insufficient resources due to a stagnated economy, displaced health workforce, and the lack of local population health data are all challenges to health system reconstruction [6]. Having an adequate health workforce becomes critical in post-conflict areas, as the availability of healthcare and accessibility to treatments remain priorities in addressing health needs. Prior to the COVID-19 pandemic, the World Health Organization (WHO) estimated a global health workforce shortage of almost 18 million by 2030, which primarily affects low-income and lower-middle-income countries [7]. The second “pulse survey” conducted by the WHO found that 89% of 135 countries reported disruptions to essential health services due to the shortage of medical resources [8].

While the pharmacy workforce is the third largest healthcare professional group worldwide (after medical doctors and nursing personnel), it is the professional group whose shortfall has not received the same level of attention in low-income countries. Over 90% of low-income countries have fewer than 5 pharmacists per 10,000 population [7], compared an average of more than 8 pharmacists per 10,000 inhabitants among the Organization for Economic Cooperation and Development (OECD) countries [9]. The health needs in conflict areas are prominent and the needs of pharmacy services are difficult to predict. The demands of technical expertise, human resources strategic planning, and service improvement are surging as the quality of health response is expected to improve. Existing work has identified an insufficient workforce and poor human resource management as key barriers to health systems [10]; it has also revealed the challenging features of immediate post-conflict periods for health service provision [6]. These conditions, acting in synergy, restrict the recovery and growth of health systems in sub-Saharan Africa [11,12,13]. While previous global pharmacy surveys successfully included almost half the member states in the WHO Africa Region [14,15,16], the current pharmacy workforce landscape remains unclear in many post-conflict regions where they are facing these challenges.

This study aims to describe the experience of the provision of pharmaceutical care services in conflict-affected areas, and to understand the challenges and barriers in the recruitment, training, and retention of the pharmacy workforce in selected sub-Saharan African countries. This study also explores the way forward for policies that facilitate a sustainable pharmacy workforce. This report constitutes one of the case studies in the WHO funded project “Health work-force development strategy in Health EDRM: evidence from literature review, case studies and expert consultations” [17].

## 2. Materials and Methods

### 2.1. Study Design

This was a qualitative study with semi-structured interviews with key informants conducted from June 2020 to February 2021. The study targeted four sub-Saharan African countries including the CAR, the DRC, Ethiopia, and South Sudan. This study focused on one of the aspects highlighted in the Sendai Framework for Disaster Risk Reduction: enhancing disaster preparedness for effective responses and to “Build Back Better” in recovery, rehabilitation, and reconstruction [18]. In particular, this study identified challenges in pharmacy service provision and workforce development, and the push and pull factors of professional recruitment and retention, which are key to the countries’ health systems and their capacity in responding to emergencies and disasters.

### 2.2. Study Setting

As of 2021, WHO classified the DRC and South Sudan as countries in Grade 3 emergencies (Grade 3 emergencies having the most substantial public health impact), and the CAR and Ethiopia as countries in Grade 2 emergencies [19]. The grading system is based on the level of public health consequences and required response from the World Customs Organization (WCO) and/or WHO. These countries were selected due to their context in the aftermath of recent wars and their prolonged state of instability due to ongoing minor conflicts. The CAR, the DRC, Ethiopia, and South Sudan ranked 188, 179, 173, and 186, respectively, in the Human Development Index in 2019 by the United Nations Development Program (UNDP) [20]. See the supplementary file (Supplementary File S1) for a brief description of the conflict in the respective countries. In summary, even though media headlines mostly focused on Ethiopia with the new round of conflicts in the northern region of Tigray and Amhara at the time of the study, the CAR, the DRC, and South Sudan all continued to suffer from insecurity and conflicts in parts of the country.

### 2.3. Study Population

Key informants were selected for their role and experience in providing pharmacy services in their respective countries [21]. Sampling in this study was purposive, aiming to maximize the variation of information [22]. Emails were sent to key pharmacy academic and training institutions in these countries, and through personal connections of the authors. Respondents fulfilling the inclusion criteria (Table 1) were recruited to participate in the study. Ethics approval was obtained from Chinese University of Hong Kong Survey and Behavioral Ethics (Reference No. SBRE-19-603). Verbal consent was obtained from all participants.

### 2.4. Data Collection

The semi-structured interviews followed a set of open-ended questions in the interview guide (Supplementary File S2), which covers four areas: respondent’s information, pharmacy services, pharmacy workforce, and sustainability and resilience. During the interview session, participants were encouraged to share not only their personal experience, but also their observations in the field [23]. All interviews were conducted by phone, Skype, or WhatsApp call, and the sessions were recorded.

### 2.5. Data Analysis

Transcripts were produced based on the audio-recording. The audio-recordings were deleted once the transcription was complete. Thematic analysis [24] was conducted: the anonymized transcripts were coded based on Grounded Theory: the codebook (Supplementary File S3) consisted of recurrent themes that emerged from the interviews and did not rely on a priori theory [25]. The analysis followed the structure of the interview guide to cover three aspects: pharmacy services, pharmacy workforce, and building a sustainable and resilience workforce.

## 3. Results

### 3.1. Characteristics of Participants

Nine interviews were conducted and the characteristics of participants are shown in Table 2. Most (7/9) of the participants were pharmacists; the remaining participants were a physician and midwife, respectively, who filled in the gap of a pharmacist’s role when there was a pharmacist vacancy.

The participants had diverse professional experience. The majority (6/9) provided care in the country of interest in either a hospital setting or mobile vaccination unit under an international NGO, others were involved in research and teaching at a university or in supply chain management in a governmental agency.

The majority (6/9) of the participants were expatriate staff, either coming from neighboring countries or from other continents. More participants reported having no experience in conflict settings in other countries (6/9) than having worked in different countries with various security contexts (3/9).

For pharmacy managers and trainers, the daily tasks and duties were described as ensuring supply of medication for service operation, ensuring quality of medications and laboratory reagents, supervising stock managers and dispensers or pharmacy technicians, training end-user units on drug management and prescribers on rational use of medicines such as antibiotics, and organizing vaccine campaigns in the case of outbreaks. Participants also described the roles of the pharmacy personnel they supervised. The daily tasks and duties performed by the stock managers and dispensers or pharmacy technicians were described as dispensing, correcting errors on prescriptions, patient medication counselling, inventory control, and monitoring stock consumption.

For researchers and lecturers, the general duties were described as teaching pharmacy degree and master’s students based on theory and their own practical experience, driving research in pharmacology and pharmacy services, promoting clinical pharmacy, and integrating pharmacy professionalism and local care contexts.

### 3.2. Thematic Analysis

Recurrent codes were grouped into 14 sub-themes, and the sub-themes were classified into 4 main themes: provision of pharmacy services, pharmacy workforce, and sustainability and resilience, and others. All the emergening codes, sub-themes and themes are presented in Table 3.

#### 3.2.1. Provision of Pharmacy Services

Pharmacy services were available through the following: hospitals and health centers operated by the government, international NGOs, or both; private medical clinics; private formal pharmacies; and local drug shops (mini-pharma, general purpose village stores, etc.). The availability and quality of pharmacy services varied by the scale of care facilities and the type of care providers. Challenges in the provision of pharmacy services can be classified into insecure-context-specific and development-context-specific challenges.

The challenges in the provision of pharmacy services under the insecure context reported by participants included logistic difficulties due to insecure roads, unexpected increases in health needs, high-tension environments, and insufficient law enforcement to ensure pharmaceutical service quality.

Participants revealed the barriers in both the provision and access of care due to insecure roads. Transport is a major component in medical logistics. When roads are insecure, there can be delays in the delivery of medical consignments and when the pharmaceutical products do not reach care facilities in time, the remaining shelf life will be shorter. Safe roads also permit people to commute and access care. According to an informant who worked in South Sudan,

“…because of the war and all this insecurity, it’s difficult for drugs to reach to the community or health facilities in time … And if the drugs are there in the facility, it’s also difficult for the community to get services, as they cannot reach to pharmacy or to hospital to get services. And so drugs expire, many drugs expire … I mean, movement is not free, most of the commodities expire before they reach to the consumers”.

(SS02)

#### 3.2.2. The Pharmacy Workforce

The participants who took part in this study, including both national and expatriate staff, filled different roles in the country of interest; these roles included pharmacy manager, trainer, stock manager, lecturer, and researcher. From the accounts of their experience in the country, it was observed that the stock manager and dispenser roles were often filled by national staff, the pharmacy manager and trainer roles were often filled by expatriate staff, and the educator and researcher roles were often filled by national staff.

Both national and expatriate staff identified that the quantity and quality of the pharmacy workforce can be a challenge. In terms of quantity, participants from all locations suggested the health needs and demands were high, while the number of pharmacy staff was low in general. Participants reported that there tended to be more pharmacy staff at larger-scale hospitals, such as teaching hospitals and specialized hospitals compared to health centers and smaller facilities, and more pharmacists in major cities compared to rural settings, but the challenge has remained and has become more significant as the number of facilities has increased over time but the number of university graduates has not caught up. This was summarized by one of the participants:

“There is lack of profession. When you compare a health center, for example, a primary hospital, a general hospital, the number of pharmacy assistant in a teaching hospital is better, but not enough… Nowadays, the number of hospitals, the number of new health centers, are increasing, especially the new hospitals. Even this makes also the challenge”.

(ET03)

The outgoing migration of skilled health workers (often referred to as brain drain) was a recurrent theme in the interviews with participants who worked in Ethiopia and the DRC, which was also attributed as one of the challenges in pharmacy workforce development. Below are excerpts from two interviews that shed light on this phenomenon:

“There are people who are even going to America you see, and Namibia … so there are people who are seeking other countries, who have more incentives to live a quality life”.

(ET03)

“There are a lot of pharmacists who leave the country, who want to go to other countries. You know that there are pharmacists in Angola, Zambia, Namibia, Zimbabwe, South Africa and even in Europe, who have been trained here. There are also Congolese pharmacists in Canada, in the United States. I must say that overall, living conditions are not very good in low-income countries, which contributes to the tendency to leave the country and go to work elsewhere. But even those who remain in the country are not very well used as I told you”.

(translated from French, DC01)

As expatriate pharmacists accounted for two-thirds of the study sample, there were unique challenges reflected by the expatriate workforce. A common theme arising from the interviews with expatriate pharmacists was that the role of a pharmacist in the post-conflict setting differed from the service model in a stable context, and such differences were compounded with differences in health systems, health needs, and pharmacy professional development in different countries. This was reflected in an example where a drug development and regulation pharmacist was deployed for a vaccination campaign:

“Because it’s not the work of a normal pharmacy, I didn’t work in the hospital or I didn’t work in the health unit, so it’s difficult to understand what you do during the intervention. So I learnt, I studied a lot a lot before I go, but I really understand after the intervention”.

(DC02)

Besides the transition from home role to expatriate role for some pharmacists, the transition of a non-pharmacy HCP’s role to a pharmacist’s role proved challenging. An example was offered by a midwife who filled the role of a medical logistician before a pharmacist was recruited for this position:

“If the medical logistician is not here and I am covering for the medical logistician, there are more needs for more explanation to be done, to the facility pharmacist, to the dispenser, properly, by a medical logistician, to tell them the proper usage of drug, the proper storage, and in case of side effect. I think there are too much gap, I will be doing the basic work, not the actual work I am [the pharmacist] supposed to do, which is a big challenge”

(SS02)

A recurrent theme in the discussion on pharmacy workforce development with the participants was “motivation”, which is pertinent at various levels in the health system. Regarding the frontline workforce, staff can be demotivated when the same amount of workload of a similar nature was rewarded by two different pay scales supported by two different institutions. It was observed in multiple locations where care facilities were co-supported by both the states and aid groups. Regarding health system development, two types of motivation issues were identified by the participants: (1) motivation from within the state to shape the development of the pharmacy profession and to regulate pharmaceutical business as well as practicing licensed pharmacists; and (2) motivation from among the pharmacy staff to expand their technical, clinical, and managerial skills.

#### 3.2.3. Building a Sustainable and Resilient Workforce

When asked about future career pathways and intentions to remain in the pharmacist’s role in the insecure context, the participants offered very different opinions.

For national staff, motivating factors would mean factors that drive them to (1) continue being in the current pharmacy profession and (2) continue staying in their home country; whereas for expatriate staff, they would mean factors that drive them to (1) continue being in the current pharmacy profession and (2) leaving home country (whether it is stable or post-conflict) to live in a post-conflict country.

For national staff, deterring factors would mean factors that drive them to either (1) seek another employment in the pharmacy profession, or (2) seek another employment outside the pharmacy profession, or (3) seek another employment (whether a pharmacy-related job or not) in another country that is less insecure than their post-conflict home country. For expatriate staff, deterring factors would mean factors that drive them to either (1) seek another employment in humanitarian medical services but outside the current organization, or (2) seek another employment outside the pharmacy profession, or (3) wish not to leave home country for any further expatriate employment.

We observed a distinct pattern of the narrated motiving factors and deterring factors for national staff and expatriate staff, respectively.

##### National Staff

The motivators for national staff to remain in their own country included aspirations to develop the pharmacy profession in their own country, foreseeable good career prospects given the experience in the country, job satisfaction from their current role, and good remuneration packages. This is reflected by the following excerpt:

“For me, I don’t mind staying in the country. And you have a lot of country-level challenges, the country has a lot of pharmaceutical challenges. But I believe I can help as a person by providing not only training but also experience from the government’s and the regulator’s perspective. I think I’m more useful staying here than going somewhere else”.

(translated from French, DC01)

The factors that drove national staff to switch to other employment included limited career advancement opportunities, low job satisfaction from non-clinical roles, and suboptimal incentives in clinical roles. One of the participants reported this general observation about the pharmacy workforce:

“But the problem is it’s not acknowledged by the government. That means it’s not recognized by the government. That means it is not incentives to retain these master pharmacists, I mean, clinical pharmacists at the hospitals. They are seeking another job”.

(ET03)

##### Expatriate Staff

The motivators for expatriate staff to continue their career in the expatriate environment in unstable countries included humanitarianism, their belief in health equity, and the opportunity to experience different cultures and to work in different health systems. Some expatriate participants also shared that serving overseas allowed them to work in different services other than being restricted to the pharmacist’s role in their home country, which is one way to build professional skills. One recurrent theme for expatriate staff retention was good experience with NGO employers—such good experience referred to the positive outcomes from the clinical activities carried out, for example, low mortality in their facility compared to the national average—as well as the positive impression about how risks were mitigated in the insecure context through vigilant planning about mobility and transparent communications about volatile situations in the country. Below are a few excerpts that showed the different motivating factors given by expatriate staff:

“I would like to stay, I like working for the humanitarian, I like working for this organization, they didn’t take any part in anything. It’s like we are here to save anyone is coming, we don’t care your position, we don’t care your religion, we don’t care what you’ve done in your life, here you are sick, I am treating you. I like these ideas”.

(CA02)

“In fact, I prefer my role as a pharmacist. And then, it allows you to know other cultures, it allows you to know the health system in another country… You see what you can bring in to improve. That’s why I prefer expatriate work”.

(translated from French, CA03)

“I mean, big motive as well as how things are organized within the organization itself. So I had good experiences so far, so I guess that is also very important what organization I’m talking about and what is the reason that we are going somewhere to be in a mission and to improve things there”.

(SS03)

The factors that deterred expatriate staff from going into overseas expatriate work again included unclear career pathways as an expatriate staff in the NGO, lack of means to develop professionally, challenges in family planning and finding an expatriate position that is suitable for family and children, and the difficulty in maintaining a livelihood in their home country while offering extended availability to the field position. The following excerpt showed an expatriate staff’s concern about the lack of professional development opportunity:

“In fact I see myself not working in the same environment. I would prefer to go back to my home country, and practice more with my field … Working in this kind of setting, I don’t improve in my career. So I would prefer to be in a place, a more organized place where I can practice more and have more knowledge on my career”.

(SS02)

## 4. Discussion

Our study identified challenges of the pharmacy services provisions from selected health facilities across the four different countries, how the pharmacy workforce had successfully or unsuccessfully coped with the service demands from conflict-affected populations, the lack of an adequate qualified workforce, and the push and pull factors for the recruitment and retention of pharmacy staff.

### 4.1. Pharmacy Service: Unpredictable Health Needs and Mismatched Pharmaceutical Supply

From a supply-and-demand point of view, the provision of health services in post-conflict areas is challenged by three factors: unpredictable health needs, unstable pharmaceutical supplies, or a mismatch of pharmaceutical supplies (products do not match the healthcare activities being carried out).

Existing studies have elucidated how conflicts enlarged the gaps in health needs among affected people. One of the priorities of health provision to conflict-affected populations used to be in the refugee camps, where epidemics and malnutrition are often the foci [26]. However, there is an increasing tendency for people to be internally displaced instead of crossing international borders [27,28]. The fuel of today’s conflicts has shifted from historical goals of state building and states’ competition for resources to seizing control of power and resources within the same state, thus forcing more people to flee their home and to seek refuge in urban areas [26,27]. Other than the conventional concerns about endemic diseases, such as malaria, cholera, and measles in overcrowded living environments, neglected tropical diseases continue to burden conflict-affected populations in some of the world’s least developed nations [29].

One major challenge identified in the provision of pharmacy services is unforeseeable health needs due to displacement or mass casualty events. Such challenges are exacerbated by the breakdown of the local healthcare systems and the supply chains of essential items, including medicines and food. Facing blockages and interruptions in the essential goods supply chain, important health interventions including vaccination campaigns and therapeutic feeding programs are inevitably hampered [28,30].

Donations have been used in humanitarian actions to ease the problems posed by pharmaceutical supply chain breakdowns; however, when the donation is not tailored to local health needs, it may cause more problems than it aims to solve. We did not identify documented mismatch of humanitarian efforts and local needs in the four selected countries, but we identified a few historical cases that happened in relevant contexts. In 1989, large amounts of donated aspirin tablets expired upon arrival in Eritrea; it took six months to burn the expired drugs. A similar example in Bosnia and Herzegovina between 1992 and 1996 saw 17,000 tons of expired drugs burned at a cost of USD 34 million [31,32]. In Sri Lanka, more than 3500 truckloads of donated pharmaceutical products arrived following the tsunami in 2004—some expired, some labelled in a language unknown to the local professionals, and some in excess quantities; eventually, 25% had to be destroyed [32]. These few well-documented cases were brought to attention due to the scale of the wastage. We should be critical about any optimism in the lack of wastage reports because wastage may escape detection when the scale is small (yet the impact can be big if the problem is a continuous one) or when there is no systematic way to evaluate wastage.

Drug expiration dates typically range from 12 to 60 months after their production. An expiration date is the date until which the manufacturer guarantees the quality of the product, and the expiration date does not mean the drug is proven to be unsafe or less effective after that date. The Shelf-Life Extension Program (SLEP) checks the long-term stability of federal drug stockpiles. Overall, 88% of 122 different drugs stored under ideal environmental conditions had their expiration dates extended by more than 1 year, with an average extension of 66 months and a maximum extension of 278 months [33]. As an example, in the situation where a patient has an infection, if a recently expired bottle of cephalexin is all that is available, one must weigh the risks versus the benefits of using it. Without proper information one is likely to throw the bottle out, whereas if the user is aware that when stored in proper conditions, cephalexin has been shown to be safe and effective for 57 months after expiration, that may significantly influence the pharmacist’s decision of allowing an infection to go untreated [33].

The challenges of appropriate allocation, storage, and information regarding unused and expired drugs remain important issues in pharmacy management. A recent study conducted in four hospitals in Ethiopia found that as much as 36.4% of anti-infective medicines and 21.4% of pain medications went to waste in the 2019 fiscal year [34]. It supports the notion that more efforts are needed in drug management and not only in drug acquisition.

### 4.2. Pharmacy Workforce—The Need for Adequate Coverage and Training

The pharmacy workforce is vital for all aspects in the medicine use process, including research and development, manufacturing, distribution, procurement, regulation, supply, pharmacovigilance, rational use, and adherence [35]. In areas affected by conflicts, a skilled and competent pharmacy workforce is more essential in order to cope with the service provision challenges mentioned previously. The task of making medicines and vaccines available requires skills in ensuring the quality of medical items delivered, minimizing wastage of drugs and medical consumables, promoting rational use of medicines by health workers, and ensuring patients’ compliance.

However, the shortage of a skilled pharmacy workforce highlighted by our study echoes the barriers to a health system that have been identified by others [10]. The global pharmacy workforce survey conducted by the International Pharmaceutical Federation (Fédération Internationale Pharmaceutique, FIP) in 2006, 2009, 2012, and 2016 [14,15,35] uncovered an alarming shortage in the pharmacy workforce in the Africa Region and in low- and middle-income countries, among which pharmacist densities were mostly below 1 per 10,000 population [14,15], which is far lower than the OECD countries’ average of 8.3 per 10,000 population [9]. The absolute growth in pharmacy workforce in the Africa Region is also the lowest among the six WHO regions; according to the FIP’s projection based on current growth trends, pharmacist densities in the Africa Region will still be less than 2 per 10,000 population by 2030 [15]. The dearth of data on the pharmacy workforce in conflict areas also limits development in this field, and a health workforce information system may contribute to mapping out local health workforce contours [36].

The post-conflict rebuilding of a health system involves three phases: the initial response to immediate health needs, restoration of health services, and restoration of the health system [37]. The shortage of pharmacy workforce in post-conflict settings may be temporarily relieved by expatriates; however, this is not a long-term solution. International actors should take capacity building into account in response planning and deliver services in a way that does not hinder health system rehabilitation [37]. Such approaches can also avoid detrimental impacts to the overall economic recovery by minimizing market distortion in the health sector [38].

Task shifting, defined as rational redistribution of tasks among health workforce teams, was observed in the settings involved in this study; for example, pharmacist’s roles were filled by non-pharmacy HCPs such as midwifes; in another example, a dispenser’s roles were filled by personnel without formal pharmacy-related training but with on-the-job training. Although the literature on task shifting in pharmacy services is scarce [39], it has been advocated and used in HIV service delivery [40]. The question of how task shifting should be recommended in terms of the pharmacy workforce in conflict settings requires a careful consideration of the following: (1) Which pharmacy services can be efficiently and effectively used in task shifting? (2) How can we recruit and provide adequate support and training for non-professional pharmacy personnel to ensure the quality of the services provided during task shifting? To help pharmacist trainers equip suitable personnel to carry out necessary tasks in pharmacy services, further research and evidence-based recommendations will be invaluable for the identification of common skill gaps and building blocks for a pharmacy task-shifting training manual.

### 4.3. Building a Sustainable and Resilient Workforce—Barriers in Talent Retention and Growth

Health workforce “brain drain”, defined as the migration of health workers from conflict-affected areas to more stable countries for shelter and employment, or to more developed countries for a better quality of life, is an often-denounced challenge [41,42]. Cometto and colleagues proposed policy solutions for countries who suffered from brain drain. These solutions included the following: (1) emphasize non-wage retention strategies (such as improving working and living conditions), (2) harness the potential of non-physician clinicians and community health workers, and (3) encouraging migrant health workers to return to their home country [41]. One of the proposed global policy solutions was the mandatory cost sharing by countries hiring from resource-poor countries as an attempt to mitigate the impact of brain drain on resource-poor countries [42]. Another mechanism for tackling brain drain is to draw on the capacity-building potential from expatriate staff. The WHO’s guide to health workforce development in post-conflict environments highlights the fact that the expatriate community has a wealth of expertise and their input, even if it is short-term counsel, which could offer some support to ministries of health to reconstruct and redevelop their health services [43].

Besides tackling the outflux of the local health workforce, a longer-term solution to expand the pharmacist talent pool would be investment in education and professional training. It should be emphasized that the quality of education should not be compromised in the course of increasing the number of schools and training programs [43]. To re-establish teaching and training standards, it is important to first strengthen the capacity of the teachers and trainers, and to redevelop the curricula [43]. For countries that are impacted by prolonged conflicts, it is also important for more advanced training programs, such as pharmacy technician diplomas or university degrees in pharmacy, to be developed in collaboration between donors and the health authority so that the training programs are competence-based programs and use objective evaluation methods to prove that the trained personnel have acquired a good standard of knowledge and skills pertinent to the health services to be provided [43].

In our study, one of the identified push factors that drives the pharmacy workforce away from conflict-affected areas was safety concerns. The concern about the safety of the health workforce in unstable areas has grown in recent years as humanitarian space shrinks [44,45,46]. The WHO has recorded over 300 confirmed attacks on healthcare facilities in fragile states in 2020 [47], whereas the Safeguarding Health in Conflict Coalition (SHCC) reported over 50 attacks almost every month from November 2020 to April 2021 [48]. While continuously tracking and reporting attacks on healthcare, the international community and local health authority must condemn such attacks and uphold the International Humanitarian Law (IHL) [49]. The experience of community engagement in Nigeria identified three good practices to protect healthcare from attacks: (1) involving local communities as an element of healthcare delivery, (2) involving community leaders as early as possible in deciding whether or not to set up a project or care facility in the area, and (3) choosing not to know affiliations to ensure equal access to healthcare for all parties [50]. One cannot be certain whether the strategy in Nigeria can be transferred to the four selected countries unless local studies offer us evidence; however, it is one example to improve local and international health workforce engagement and may have referencing value for countries facing similar challenges.

### 4.4. Limitations

Due to the difficulties of reaching participants in unstable contexts, especially during the COVID-19 pandemic, and the continued challenging situations for those intended participants, the study sample size was small (*n* = 9) and the majority of the recruited participants were expatriate pharmacists. This has limited our ability to generalize our findings across all health facilities and across different regions of the selected countries. Furthermore, due to the limitations from the representation of the respondents, limited information from the leadership and governance level from the Ministry of Health was solicited. Our study, however, provides important perspectives from the pharmacy workforce in these conflict-affected regions, and provides some insights into the need for further studies and solutions that may be needed to overcome the constraints in achieving universal health coverage.

## 5. Conclusions

This study described how pharmaceutical services were delivered in selected conflict-affected areas in sub-Saharan African and detailed some conflict-setting-specific challenges encountered by the participating pharmacy staff. Policy solutions on national and international levels are urgently required and efforts that can bring about long-term improvements should be prioritized. These include publishing best practice recommendations in drug management in the conflict settings, establishing information systems to monitor the need and the capacity of pharmacy services, future research on the optimal use of task shifting for pharmacy workforce, re-establishing pharmacy teaching and training standards including a pharmacy curriculum for conflict settings, emphasizing retention strategies including stable income and safe working conditions, and promoting the IHL and the protection of health workers. This is essential to achieve universal health coverage and the targets of the sustainable development goals for the conflict affected areas, as well as to “leave no one behind”.

## Figures and Tables

**Table 1:** Inclusion and exclusion criteria for key informant recruitment for semi-structured interviews.

Inclusion Criteria
(1) Have worked in one of the following pharmacy capacities in the selected country for at least 3 months: Pharmacy strategic planning; Pharmaceutical product supply chain management; Rational use of medicine (e.g., antibiotic stewardship program); Patient counselling and/or treatment compliance assessment; Clinical pharmacy services (e.g., ward rounds, case management); Dispensing and/or extemporaneous preparation of medication; Pharmacovigilance; Regulatory affairs and legal compliance. (2) Are able to communicate effectively in English or French. (3) Have given informed consent verbally during the audio-recorded interview.
**Exclusion Criteria**
(1). Informed consent verbally not given during the audio-recorded interview, or was withdrawn during or after the interview. (2). Communication was not effective due to language barrier or technical difficulties, such as poor phone connection.

**Table 2:** The characteristics of participants.

Total		*n* = 9
Country	The Central African Republic	2
	The Democratic Republic of Congo	2
	Ethiopia	2
	South Sudan	3
Professional Background	Pharmacist	7
	Physician	1
	Midwife	1
Years of Experience (range: 6–36, median: 10)	5 to <10 years	3
	10 to <20 years	4
	20 years and above	2
Experience in other conflict settings	Yes	3
	No	6
Sector	Government	1
	NGO Hospital	6
	University	2
Staff Category	Expatriate	6
	National	3
Sex	Female	5
	Male	4
Age	20–29	1
	30–39	4
	40–49	3
	50–59	1

Regarding the roles and duties of the participants, the reported roles included pharmacy manager, trainer, researchers, and lecturers.

**Table 3:** The emerging themes from interview transcripts.

Themes	Sub-Themes	Associated Codes
Provision of pharmacy services	Provider	Government/NGO/Private/Religious group
	Type	Health center/Hospital/Locum/Mobile/Traditional healer
	Barrier	Cost/Daily quota/Foreign provider/Free
	Good	Comprehensive range of service/Inventory control system/Motivated staff/ Practice according to protocol/Proper medication storage condition/ Staff with good knowledge/Sufficient supervision to identify errors
	Bad	Frequent shortage of drugs/Insufficient equipment/ Insufficient counselling to patients/ Little documentation/Long waiting time/Medication errors/ Regulation regarding drug product quality/Regulation regarding profession/Staff’s skill not matching task requirement/Unmotivated staff
	Conflict-specific	Service interruption due to volatile security context/ Shortage of local pharmacy staff due to brain drain/ Shortage of local pharmacy staff due to education interrupted by conflicts/ Transport difficulties due to insecure roads/ Unforeseeable health needs due to displacement/ Unforeseeable health needs due to mass casualty events
	General	Communication with direct manager with non-pharmacy profile/ Distribution of medication outside scope of care/ Global shortage of pharmaceutical product/ Power shortage and cold chain breakdown
Pharmacy workforce	Pharmacist Role	Consumption forecast and supply strategy/Consumption unit staff training/ Drug inventory management/Evaluation of distribution system performance/ Good distribution practice compliance/Inpatient consultation/ Pharmacy staff training/Promotion of rational use of medicine/ Research and teaching/Vaccine campaign for outbreak control
	Filled by Who	National/Expatriate staff; Pharmacist/Physician/Midwife/Nurse; Community health worker
	How Recruited	Central recruitment via government posting/ Central recruitment via NGO posting/ Task shifting due to operational need
Sustainability and resilience	Retention Motivator	Contribution to country profession development/Humanitarianism/ Opportunity for more diverse work than a career in expatriate’s home country/ Positive experience with current post and organization/Satisfactory remuneration
	Retention Barrier	Availability and expatriate mission duration mismatch/ Family planning incompatible with high insecurity expatriate mission/ Long working hours with unsatisfactory remuneration/ Preference for more clinical role/Unclear career prospect
Others	Positive Note	National staff returning from education in foreign countries/ Organization’s coordination mechanism regarding security/ Staff’s eagerness to learn/Supported by direct manager/Trust in team
	Negative Note	Different sense of normality/Hostility against HCP/ Individual demotivation/System inertia
	General Description	Gradient in workforce/Service availability and quality across country

## Data Availability

Due to the nature of this research, participants of this study did not agree for their data to be shared publicly, so supporting data is not available.

## Supplementary Materials

The following are available online at https://www.mdpi.com/article/10.3390/ijerph18158132/s1, Supplementary File S1: A brief description of the conflict background in the four countries, Supplementary File S2: Interview Topic Guide, Supplementary File S3: Codebook.
