# Supplementary material for: Filling the Gaps in the Pharmacy Workforce in Post-Conflict Areas: Experience from Four Countries in Sub-Saharan Africa
Source: Int J Environ Res Public Health. 2021 Jul 31;18(15):8132. doi: 10.3390/ijerph18158132 (PMC8346012; doi:10.3390/ijerph18158132)
Supplement: Supplementary file 1 [file ijerph-18-08132-s001.zip › Supplementary File S1.pdf]

A brief description of the conflict background in the four countries

1. Central African Republic (CAR)

The conflict in CAR started between the Séléka coalition and the anti-balaka militias in the end of 2012[1,2]. As the clashes between the Séléka coalition and the anti-balaka militias continued, the country plummeted into chaos and violence. In 2014, a transitional government was formed[1,3]. To date, the conflict between ex-Séléka and anti-balaka members has not ceased completely, but continue in the form of intercommunal clashes between pastoralist and farming communities in the rural areas[2].

2. Democratic Republic of Congo (DRC)

The First Congo War started as civil war between armed groups in Zaire (present-day DRC) in 1996. The situation further destabilised as the Rwandan army seized Kinshasa and replaced the Zairean president with the rebel group leader; and the country was renamed the Democratic Republic of Congo. The Second Congo War started in 1998 and ended in 2003 as the transitional government was sworn in to office[4]. The International Peace Information Service (IPIS) has mapped the conflict motives of the four major armed groups in its 2008 report[5]; the presence of armed groups, the involvement of illegal trades, and the conflicts around mining areas continued to preclude security and stability in the eastern region of DRC.

3. Ethiopia

The conflict in Ethiopia's Somali Region dates back to 1948, when Ethiopia gained authority over the Ogaden area from the British government[6]. The Ogaden war, also known as the Somali-Ethiopian war, took place from 1977 to 1978[7]. The Ogaden area was destabilised again in 2007: the Ogaden National Liberation Front (ONLF) and the Ethiopian government clashed frequently until a peace agreement was signed in 2018[8]. On the other hand, the tension among the three major ethnic groups, Oromo, Amhara and Tigrean, escalated during the study period, and new conflicts were reported in the Tigray area as well as in Amhara region[9–11].

4. South Sudan

Following the long-standing conflicts between the ethnic African rebels in southern Sudan and the Arab-dominated government in the North, a peace deal was made in 2005, making ways for the referendum in 2011, in which 99% voted for the separation of southern Sudan from the North[12,13]. The southern Sudan thus gained independence and was named as South Sudan. As the negotiation between South Sudan and Sudan over sharing of oil revenue and debt continued, the tribal conflicts inside South Sudan added more challenges to the young nation's path to stability and development[13].

Reference

1. Conciliation Resources. History: Central African Republic conflict. Accessed March 22, 2021. http://148.251.69.135/where-we-work/east-and-central-africa/history-central-african-republic-conflict-0
2. Crisis GroupCrisis Group. (2014). The Central African Republic's Hidden Conflict. https://www.crisisgroup.org/africa/central-africa/central-african-republic/central-african-republic-s-hidden-conflict. The Central African Republic's Hidden Conflict. Published 2014. Accessed March 22, 2021. https://www.crisisgroup.org/africa/central-africa/central-african-republic/central-african-republic-s-hidden-conflict
3. Radio France Internationale. Catherine Samba-Panza, nouvelle présidente de Centrafrique: pourquoi elle. Published 2014. Accessed March 22, 2021. https://www.rfi.fr/fr/afrique/20140120-centrafrique-catherine-samba-panza-femme-dialogue
4. Human Rights Watch. DR Congo: Chronology. Published 2009. Accessed March 22, 2021. https://www.hrw.org/news/2009/08/21/dr-congo-chronology#_Independence
5. Spittaels S, Hilgert F. Mapping conflict motives: Eastern DRC. *Antwerp IPIS*. Published online 2008:1-44. http://ipisresearch.be/maps/Oost-Congo/20080506_Mapping_Eastern_DRC.pdf

6. The University of Central Arkansas. Dynamic Analysis of Dispute Management (DADM) Project: Ethiopia/Ogaden (1948-present). Accessed May 2, 2021. https://uca.edu/politicalscience/dadm-project/sub-saharan-africa-region/69-ethiopiaogaden-1948-present/
7. Human Rights Watch. War Crimes and Crimes against Humanity in the Ogaden area of Ethiopia's Somali Region. Accessed May 2, 2021. https://www.hrw.org/report/2008/06/12/collective-punishment/war-crimes-and-crimes-against-humanity-ogaden-area
8. Abdi A. One year on: moving from war to peace in Ethiopia. Published 2019. Accessed May 2, 2021. https://www.c-r.org/news-and-insight/one-year-moving-war-peace-ethiopia
9. Environmental Systems Research Institute (Esri). The Oromo Conflict in Ethiopia and the formation of the OLF. Accessed May 2, 2021. https://www.arcgis.com/apps/MapJournal/index.html?appid=c998179af6fe468c810fdc30bf869bbd
10. Al Jazeera. Ethiopia's Tigray conflict explained in 500 words.https://www.aljazeera.com/news/2020/11/10/ethiopias-tigray-conflict-explained-in-500-words. Published November 20, 2020. Accessed May 2, 2021.
11. Reuters. Eighteen killed in clashes between Ethiopia's Oromo, Amhara groups.https://www.reuters.com/world/africa/eighteen-killed-clashes-between-ethiopias-oromo-amhara-groups-2021-04-19/. Published April 20, 2021. Accessed May 2, 2021.
12. Boswell A. Deadly Clashes on South Sudan's Path to Freedom.http://content.time.com/time/world/article/0,8599,2048788,00.html. Published February 12, 2011. Accessed May 2, 2021.
13. BBC News. South Sudan becomes an independent nation.https://www.bbc.com/news/world-africa-14089843. Published July 9, 2011. Accessed May 2, 2021.
