# Supplementary material for: Filling the Gaps in the Pharmacy Workforce in Post-Conflict Areas: Experience from Four Countries in Sub-Saharan Africa
Source: Int J Environ Res Public Health. 2021 Jul 31;18(15):8132. doi: 10.3390/ijerph18158132 (PMC8346012; doi:10.3390/ijerph18158132)
Supplement: Supplementary file 1 [file ijerph-18-08132-s001.zip › Supplementary File S2.pdf]

Interview Topic Guide

A. Respondent's Information

Staff category	National / Expat
Sector	Local government / Local NGO / Intl. NGO / Private
Field position	
Period in the field	
Current position	
Total years of experience	
Gender ; Age	F/M/T/X; 18-29/30-39/40-49/50-59/60-69/>70
Nationality	
Current location	
Date of interview	
Informed consent (verbal)	

1) Can you describe your role and years of experience in pharmacy services in your home country?
2) What about your role and experience in the country/ region of our current discussion?
3) Have you worked in other post conflict settings before?

B. Pharmacy Service

1) Availability (personal experience): Can you briefly describe how people access the pharmacy service you provide in the country of discussion?
2) Quality (personal experience): What do you think about the quality of health care in your work place?
3) Availability (general observation): Are there other pharmacy service available in this country? How do people access them?
4) Quality (general observation): What do you think about the quality of health care in the other providers that you have just mentioned?
5) Drawing from the experience of your work and your observation about other pharmacy services in this country, what would you say are the challenges? What should be kept? What should be changed?

C. Pharmacy Workforce

1) Recruitment: What did you do before taking up this field position? How did you learn about this opportunity and how did you get recruited? What was the required profile on the vacancy notice that you saw? How does it compare to the profile you had at the time of recruitment?
2) Training: Did you have any training before taking up this role? Has your workplace offer any training for this role? Do you feel that the training and support you have received are useful and/or sufficient? What more training would you want to receive in the future?

D. Sustainability & Resilience

1) Retention: Where do you see yourself in 3-5 years? What motivates you to stay or leave the role / career in this field?

2) We do hope that this country will continue its path in an increasingly stable and secure context now that the major conflicts are over, but as minor conflicts are still ongoing / political instability is still present, what do you think the health care system or more specifically, the pharmacy service providers can do to prepare for any emergency situation?
3) What advice would you give your government / health policy decision makers / international stakeholders / the fellow colleagues in this field, if any?
