# Supplementary material for: Filling the Gaps in the Pharmacy Workforce in Post-Conflict Areas: Experience from Four Countries in Sub-Saharan Africa
Source: Int J Environ Res Public Health. 2021 Jul 31;18(15):8132. doi: 10.3390/ijerph18158132 (PMC8346012; doi:10.3390/ijerph18158132)
Supplement: Supplementary file 1 [file ijerph-18-08132-s001.zip › Supplementary File S3.pdf]

Codebook

Service.Availability.Barrier

Part A Q1 - pharmacy service availability

Workforce.Who

Part B Q1 - Who is offering pharmacy services?

Sustainability.Retention.Yes

Part C Q1 - Will you stay in this field in the next 3-5 years? Why or why not?

Service.Availability.Type.Hospital

Part A Q1 - pharmacy service availability

Service.Quality.Good

Part A Q2 - pharmacy service quality

Service.Challenge

Part A Q3 - Challenges in providing pharmacy services in the field

ConflictSpecific

General classification of code

Service.Quality.Bad

Part A Q2 - pharmacy service quality

Workforce.How

Part B Q2 - How did you get recruited or involved in this pharmacy role?

Workforce.What

Part B Q3 - What former experience, training and support did you have?

Workforce.Challenge

Part B Q4 - What are the challenges you observed in the pharmacy workforce in this country?

Sustainability.Retention.No

Part C Q1 - Will you stay in this field in the next 3-5 years? Why or why not?

Strategies.Observed

What are the good practices or strategies that you have observed that can be useful for countries in a similar context?

OtherExp.Volunteer

Other experience in conflict / post-conflict settings (volunteering)

OtherExp.Mission

Other experience in conflict / post-conflict settings (contract-based)

PharRole

Description of the role & duty as a pharmacist in the country.

Service.Availability.Type.HealthUnit

Part A Q1 - pharmacy service availability

Service.Availability.Free

Service.Availability.Type.Locum

Part A Q1 - pharmacy service availability

Service.Availability.Cost

Provider.NGO

Provider.Government

Provider.Private

Service.Availability.Type.Mobile

Part A Q1 - pharmacy service availability

Strategies.Imagined

What are the good practices or strategies that you think might be useful for countries in a similar context?

PositiveNote.Competent&Learn

Competency and eagerness to learn observed in subordinates / other health care staff

PositiveNote.Trust

PositiveNote.Prep

Pre-deployment preparation

PostiveNote.Understanding

Humanitarianism

Gradient

Within country differences due to conflict hotspot distribution and urban vs. rural settings.

Strategies.Observed.Preparedness

PositiveNote.Coordination

Coordination in humanitarian care provision observed among actors

NegativeNote.Hostility

Local community / health workers / armed group / patient's family showing sentiment or hostility against foreign health care staff

NegativeNote.Mentality

Mentality of subordinates / other health care staff

Service.Availability.Type.Traditional

Provider.Religious

PositiveNote.Conscience
